# Factors associated with the resilience of Tibetan adolescent survivors five years after the 2010 Yushu earthquake

**DOI:** 10.1371/journal.pone.0231736

**Published:** 2020-04-23

**Authors:** Ying Lu, Dongliang Yang, Ying Niu, Huaguo Zhang, Bingli Du, Xiaolian Jiang

**Affiliations:** 1 Institute for Disaster Management and Reconstruction, Sichuan University-The Hong Kong Polytechnic University, Chengdu, Sichuan Province, People’s Republic of China; 2 Cangzhou Medical College, Cangzhou, Hebei Province, People’s Republic of China; 3 West China School of Nursing/West China Hospital, Sichuan University, Chengdu, Sichuan Province, People’s Republic of China; University of Memphis, UNITED STATES

## Abstract

Resilience contributes to the recovery of disaster victims. The resilience of Tibetan adolescents after the Yushu earthquake has not been properly studied. This study aimed to examine the current resilience and associated factors in Tibetan adolescent survivors in the hardest-hit area 5 years after the Yushu earthquake. This cross-sectional survey was conducted in the area hit the hardest by the Yushu earthquake. Data were collected from 4681 respondents in October and November 2015. Measurements included the participant characteristics, traumatic earthquake experience, the Connor-Davidson resilience scale (CD-RISC), and the social support appraisals (SS-A) scale. The individual datasets were randomized as 80% for the training set and 20% for the validation set. The mean resilience score of the Tibetan adolescent survivors was 55.0±12.3. Thirteen variables were entered into the regression equation. The three dimensions of social support (from family, from friends, from others than family/friends) were positively associated with resilience (all P<0.05), among which support from others than family/friends was the strongest (r = 0.388, P<0.001). Academic performance, activeness of participation in school activities, harmonious relationship with teachers/classmates, health over the last year, and regular physical exercise were positively associated with resilience (all P<0.05). Being female and being extremely worried about their own lives were negatively associated with resilience (both P<0.05). In conclusion, among Tibetan adolescent survivors to the Yushu earthquake of 2010, support from others than family/friends was the strongest positive factor associated with resilience, while being female and extreme worry about their own lives were negative factors. These results expand our knowledge regarding resilience in Tibetan adolescent disaster survivors.

## Introduction

China is an earthquake-prone country. From 2004 to 2018, 191 earthquakes occurred in China, of which 37 were above magnitude 6.0. Together they were responsible for 73,176 deaths and 486,231 injured [1]. At 7:49 am on April 14, 2010, a 7.1-magnitude earthquake struck Yushu, China, causing 2698 deaths and 12,135 injured. The hardest-hit area was Gyegu town, the government seat of Yushu Tibetan Autonomous Prefecture, located at >4000 meters altitude, and where Tibetans account for approximately 97% of the population [2]. Natural disasters result in casualties and physical/material damage and also threatens the mental health of the survivors [3,4]. Traditionally, studies have focused on negative sequelae such as post-traumatic stress disorder (PTSD) [3], but the role of resilience as a protective factor for an individual's mental health and quality of life is being recognized [3,5]. Resilience is generally defined as a dynamic process of effective adaptation after exposure to adversity [6,7], but there are debates on what constitutes resilient behavior [8]. An individual's ability to “thrive under adversity” can be used as an indicator to assess the outcomes of trauma-related psychiatric disorders [9,10]. In the present study, we defined resilience as the personal ability to adapt to traumas, stressors, or adversity effectively.

The factors influencing resilience are usually divided into risk and protective factors [11]. Those factors interact with each other to influence the development of resilience [12,13]. Adolescence is a pivotal period of life marking the transition from childhood to adulthood, marked by hormonal changes, gaining guardian independence, risk-taking, emotional reactivity, and important changes in the environment [14]. Puberty is a sensitive period for traumatic events, and it may be associated with the development of psychiatric diseases in adulthood [15]. Traumatic events or serious disasters (e.g., earthquakes and wars), long-term adverse social environment, and pressures and tasks related to individual development at the mid-late stages of adolescence are considered as possible risk factors for adolescent development [16]. Previous studies have found that earthquake exposure and secondary negative life events after an earthquake are risk factors for resilience [17,18], but a positive correlation between traumatic experience and resilience has been shown [19]. Indeed, Scali et al. [19] indicated that traumas could induce some "vaccination effect", meaning that traumatic events could modify the self-evaluation of resilience, making the individuals stronger when facing adversity [19,20]. Protective factors for resilience in Western cultures around the globe include constitutional resilience (abilities of constitutions to cope with attacks and in the end to cope with a real crisis [21]), optimism, finding purpose in life, internal locus of control, strong self-efficacy, sociability, academic achievement, personal attributes (like high awareness of the situations, of one's own emotions, and others' behaviors), supportive families, school experiences, supportive communities, socioeconomic status [22–25], child intelligence quotient [26], parental education level, parenting style [27], parent-child relationships [26,28], relationships with teachers or classmates [29], intervention projects through the schools [30], and supportive mentors [31]. Previous studies have also found that resilience is situational, i.e., that the protective and risk factors of resilience will change according to different situations and will result in different outcomes [32].

It is known that child, and adolescent survivors are a psychological stress-susceptible population to external adverse events. Xiao et al. [33,34] showed that Yushu child and adolescent survivors suffered from different levels of psychological problems, and the incidence of PTSD was 24.4% in the three years after the Yushu earthquake. Unfortunately, previous studies focused on the negative psychological problems experienced by child and adolescent survivors [35–37] and resilience was barely mentioned. In addition, in these studies, the participants were the students who moved to other areas to go to school for two years after the earthquake.

The Yushu earthquake area is situated in the relatively remote and undeveloped plateau mountainous area in western China, where the Tibetan culture is predominant. Five years after the earthquake, the current state of resilience among Tibetan adolescent survivors, given their unique geographical, cultural, social, and economic environment, is unknown, and the factors that have affected their resilience are still unidentified. Therefore, the aim of the present study was to examine the current resilience status and identify the associated factors of resilience in Tibetan adolescent survivors in the hardest-hit area 5 years after the Yushu earthquake. The results might provide useful insights for the post-disaster management and counseling of adolescents. Eventually, such data could allow better tailoring of the counseling to each individual.

## Methods

### Study design and participants

This was a cross-sectional study. The participants were recruited from four secondary schools (grades 7–12) in Gyegu town in October and November 2015. Gyegu town was almost completely destroyed by the earthquake. All participants met the following inclusion criteria: 1) Tibetan; 2) personally experienced the 2010 Yushu earthquake; 3) secondary school student of 12–18 years of age; and 4) willing, along with his/her guardians, to sign the written consent form for voluntary participation. Individuals with cognitive impairment or problems with hearing, verbal, and/or written communication were excluded. Ethical approval was provided by the Human Subjects Ethics Sub-committee of Sichuan University.

In theory, adolescence is the transitional period between childhood and adulthood, usually between the ages of 12 and 22. In China, much of the research on Chinese adolescents has focused on the traditional middle school years, which are 12 to 18 years old, according to China's legal school age. Anyone over 18 is considered an adult. The main social place of Chinese middle school students is school, and their main social role is to study, and they have little contact with society. Those characteristics change dramatically after middle school since they begin working or go to university, which is considered in China as adult age. Therefore, the age of adolescents in this study is defined as middle school students aged 12–18 years; this definition is also used in other studies from China [38–40].

### Questionnaire survey

The survey was conducted approximately five years after the disaster. Four parts were included: demographic and injury-related data, traumatic earthquake experience, Connor-Davidson resilience scale (CD-RISC), and Social support appraisals (SS-A) scale. The questionnaire was administered in Chinese, as Chinese is the language of instruction in schools in the Yushu area. Prior to the survey, we gained the permission and assistance of the local education management departments, as well as of the principals and class teachers of the schools. The participants were asked to complete a self-administered questionnaire in their classroom with the assistance of the researchers, who were two doctoral students and a psychology professional. All three researchers had received training for data collection (including instructions for filling the questionnaire, meaning and filling requirements of each item in the questionnaire, on-site check whether there was an omission, and handling methods after collection). Researchers collected the questionnaires after checking each one for completeness. If a questionnaire was incomplete because of negligence, it was returned to the participant to be completed.

Prior to the investigation, we explained the study purpose to the participants, assured them of the confidentiality of their personal data and the contents of the questionnaire, and obtained the consent of the participants and legal guardians. In the course of the investigation, the right of withdrawal was fully respected, and attention was given to the protection of the privacy of the participants during the entire research process. In each school, there were teachers and psychological professionals responsible for dealing with the psychological discomfort that the study may have caused. Non-research team members were not permitted to access the original data. In place of each participant’s name, a code was used for data entry and analysis.

### Demographic and injury-related data

Data included age, sex, single-child status, left-behind child (the father or mother has not lived with the child for more than half a year due to reasons such as going out to work), parental education level, relationship with family members, family financial conditions, living conditions, activeness of participation in school activities, academic performance (according to the results of the school’s last academic year), academic stress, relationship with classmates, relationship with teachers, health over the last year, whether they undertook regular physical exercise (three or more times a week, each time lasting 40 min or more), whether they had experienced stressful events (events that occurred in the past or persisted, caused or are causing moderate or above distress, other than earthquakes and academic stress; e.g., parents often quarreling with each other; oneself being punished), and whether they had received psychological counseling.

### Earthquake traumatic experience

Each participant’s experience of the traumatic earthquake was assessed using twelve items: whether they were buried in the earthquake; whether they were injured/disabled in the earthquake; whether they had family members/classmates/teachers who died in the earthquake; whether they experienced severe property loss; whether they witnessed someone else being buried/injured or dying; whether they were extremely worried about their own lives; whether they were extremely worried about the health of family and friends; and whether they felt guilty because someone else was killed or injured in the earthquake.

### Connor-Davidson Resilience Scale (CD-RISC)

The CD-RISC was used to assess the level of individual resilience. The scale was developed by Connor and Davidson [41] and has been validated among Chinese children and adolescents [42,43]. Fu et al. [42] validated the questionnaire in child and adolescent survivors of the 2008 earthquake in China. Yu et al. [43] validated the questionnaire in adolescents after the 2008 earthquake in China. The scale includes five dimensions: 1) personal competence, high standards, and tenacity; 2) trust in one’s instincts, tolerance of negative affect, and strengthening effects of stress; 3) positive acceptance of change, and secure relationships; 4) control; and 5) spiritual influences. The scale is composed of 25 items, which are rated on a 5-point scale, with answers ranging from 0 = “not true at all” to 4 = “true nearly all the time.” The total possible score ranges from 0 to 100, with higher scores corresponding to higher resilience. Cronbach’s α for the CD-RISC in our study was 0.89.

### Social Support Appraisals (SS-A) scale

Social support was evaluated using the SS-A scale developed by Vaux et al. [44] and revised by Xin et al. [45]. The scale was used to assess the adolescents' perceived respect, trust, love, care, and other support from family, friends, and others than family/friends. It comprises 20 items in three subscales: support from family, friends, and others than family/friends. Each item is rated on a 5-point Likert scale, ranging from 1 “never” to 5 “always.” Each subscale score was calculated by averaging the scores for all items belong to it. The higher the score, the more social support a participant believes they have. In our study, the Cronbach’s α coefficients for the whole scale, and the three subscales (support from family, friends, and others than family/friends) were 0.88, 0.80, 0.82, and 0.79, respectively.

### Statistical analysis

Statistical analysis was performed using R software version 3.5.1 and SPSS 19 (IBM Corp., Armonk, NY, USA). The level of significance was set at 0.05 (two-tailed) for all statistical inferences.

Participant characteristics and traumatic earthquake experiences were expressed as numbers (%). CD-RISC scores and SS-A scores were expressed as means and standard deviations. Pearson’s correlations were calculated to evaluate the potential relationships between the CD-RISC and SS-A subscales.

The data were randomly divided into two parts, 80% as the training set and 20% as the verification set. First, using data from the participants included in the training set, the least absolute shrinkage and selection operator (LASSO) method was used to select the optimal associated features among the potential factors of resilience in Tibetan adolescent survivors. Features with non-zero coefficients in the LASSO regression model were selected [46]. Second, always using the data from the training set, a multiple stepwise linear regression analysis (α_in_ = 0.05, α_out_ = 0.10) was used to build a prediction model for resilience by incorporating the features selected in the LASSO regression model. Finally, the linear regression model was validated in the validation data set.

## Results

### Characteristics and earthquake traumatic experience of the participants

A total of 5508 participants met the inclusion criteria, and 565 individuals from the four participating schools declined to participate. A total of 4943 questionnaires were administered, and 4681 (94.7%) valid questionnaires were collected. The mean age of the participants was 15.7±1.8 years, and 2466 (52.7%) were female. Among the participants, 6.9% were burried, 14.6% were injured, 0.9% were disabled, 17.4% had family members who died, 33.9% had classmates or teachers who died, 42.9% experienced severe property loss, 36.9% witnessed someone else being buried, 62.9% witnessed someone being injured, 44.7% witnessed someone dying, 64.2% were extremely worried about their own life, 84.8% were extremely worried about the health of their family and friends, and 80.0% felt guilty because someone else was killed or injured. The participant characteristics and traumatic earthquake experience are shown in Tables 1 and 2, respectively. The CD-RISC scores were higher in those who were injured (P = 0.009), experienced severe property loss (P = 0.015), witnessed someone else being buried (P<0.001), witnessed someone else being injured (P<0.001), witnessed someone else dying (P = 0.009), were extremely worried about their own lives (P = 0.022), were extremely worried about the health of family and friends (P<0.001), and felt guilty because someone else was killed or injured (P = 0.004) (Table 2).

**Table 1:** Characteristics of the participants and CD-RISC scores (n = 4681).

Variables	n (%)	CD-RISC score		P for CD-RISC score
		mean	SD	
Age (years)				0.673
≤14	1166 (24.9)	55.2	13.2	
>14	3515 (75.2)	55	12	
Sex				**<0.001**
Female	2466 (52.7)	53.9	12.3	
Male	2215 (47.3)	56.3	12.2	
Left-behind child				0.602
Yes	436 (9.3)	54.7	12.5	
No	4245 (90.8)	55.1	12.3	
Single-child				0.16
Yes	377 (8.1)	55.9	13.1	
No	4304 (92.0)	55	12.2	
Father’s education level				**<0.001**
Primary school or below	3626 (77.5)	54.6	12	
Junior middle school	516 (11.0)	55.3	12.5	
Senior middle school	233 (5.0)	56	13	
College/University or above	306 (6.5)	58.7	13.6	
Mother’s education level				**<0.001**
Primary school or below	4094 (87.5)	54.7	12	
Junior middle school	248 (5.3)	55.5	13.2	
Senior middle school	133 (2.8)	55.2	14	
College/University or above	206 (4.4)	60	13.4	
Relationship with family members				**<0.001**
Harmonious*	3848 (82.2)	55.7	12.2	
Normal	765 (16.3)	52.3	11.8	
Poor	68 (1.5)	50.5	14.2	
Family financial status**				**<0.001**
Good	535 (11.4)	58.1	13.6	
Intermediate	3280 (70.1)	54.6	12.1	
Low	866 (18.5)	54.6	12	
Living conditions				**<0.001**
Good	1000 (21.4)	57.5	13.1	
Intermediate	3302 (70.5)	54.3	11.9	
Low	379 (8.1)	54.5	12.8	
Activeness of participation in school activities				**<0.001**
Active	1187 (25.4)	59.7	11.8	
Moderate	2476 (52.9)	54.5	11.8	
seldom	1018 (21.8)	50.9	12.2	
Academic performance				**<0.001**
Excellent	145 (3.1)	62.7	12.5	
Good	1843 (39.4)	57.7	12.3	
Medium	2418 (51.7)	53.1	11.6	
Poor	275 (5.9)	50.4	12.4	
Academic stress				0.075
High	2439 (52.1)	54.6	12.2	
Moderate	2138 (45.7)	55.5	12.2	
Low	104 (2.2)	55.1	14.4	
Relationship with classmates				**<0.001**
Harmonious*	2891 (61.8)	57.1	12.1	
Normal	1741 (37.2)	51.8	11.7	
Poor	49 (1.1)	44.5	12.9	
Relationship with teachers				**<0.001**
Harmonious*	1971 (42.1)	58	12.2	
Normal	2532 (54.1)	53.2	11.7	
Poor	178 (3.8)	48.5	13.6	
Health over last year				**<0.001**
Well	1931 (41.3)	57.2	12.4	
Moderate	2490 (53.2)	53.5	11.8	
Poor	260 (5.6)	53.6	12.9	
Undertaking regular physical exercise				**<0.001**
Yes	2442 (52.2)	56.3	12.4	
No	2239 (47.8)	53.7	12	
Stressful events				0.988
Yes	2187 (46.7)	55	12.1	
No	2494 (53.3)	55	12.4	
Receiving psychological counseling				**0.014**
Group counseling	1620 (34.6)	55.4	12.6	
Individual counseling	128 (2.7)	57.6	11.9	
No	2933 (62.7)	54.7	12.1	

*Harmonious was defined as the absence of estrangement or conflict among individuals.

** Because the respondents were teenagers, they do not know the specific family income, so they are only asked to choose according to their own subjective judgment, without giving specific values.

**Table 2:** Earthquake traumatic experience and CD-RISC scores (n = 4681).

Variables	n (%)	CD-RISC score		P for CD-RISC score
		mean	SD	
Were buried in the earthquake				0.108
Yes	323 (6.9)	56.1	12.4	
No	4358 (93.1)	55	12.3	
Were injured in the earthquake				**0.009**
Yes	683 (14. 6)	56.2	12.4	
No	3998 (85.4)	54.8	12.3	
Were disabled in the earthquake				0.451
Yes	41 (0.9)	53.6	14.4	
No	4640 (99.1)	55	12.3	
Had family members who died in the earthquake				0.694
Yes	812 (17.4)	55.2	12.7	
No	3869 (82.7)	55	12.2	
Had classmates/teachers who had died in the earthquake				0.14
Yes	1585 (33.9)	55.4	12.6	
No	3096 (66.1)	54.8	12.1	
Experienced severe property loss				**0.015**
Yes	2003 (42.8)	55.5	12.5	
No	2678 (57.2)	54.7	12.1	
Witnessed someone else being buried				**<0.001**
Yes	1726 (36.9)	56	12.3	
No	2955 (63.1)	54.5	12.2	
Witnessed someone else being injured				**<0.001**
Yes	2897 (61.9)	55.6	12.4	
No	1784 (38.1)	54.2	12	
Witnessed someone else dying				**0.009**
Yes	2094 (44.7)	55.5	12.4	
No	2587 (55.3)	54.6	12.2	
Were extremely worried about their own lives				**0.022**
Yes	3003 (64.2)	54.7	11.9	
No	1678 (35.9)	55.6	12.9	
Were extremely worried about the health of family and friends				**<0.001**
Yes	3969 (84.8)	55.3	12.1	
No	712 (15.2)	53.4	12.9	
Felt guilty because someone else was killed or injured in the earthquake				**0.004**
Yes	3745 (80.0)	55.3	12.1	
No	936 (20.0)	54	12.8	

### Resilience scores

The mean CD-RISC score was 55.0±12.3 (range, 12–91). Tables 1 and 2 show the CD-RISC scores among the different characteristic groups and different earthquake traumatic experience groups, respectively.

### Correlations between resilience and social support

Table 3 shows the social support scores and Pearson’s correlations between resilience and social support. Support from family, support from friends, and support from others than family/friends were significantly and positively correlated with resilience. The correlation between support from others than family/friends and resilience was the strongest of all of these relationships (r = 0.388, p<0.001).

**Table 3:** Social support and correlations between resilience and social support (n = 4681).

SS-A subscales	Mean	SD	Correlation with resilience	
			r	p
Support from family	4.0	0.6	0.279	**<0.001**
Support from friends	3.7	0.6	0.309	**<0.001**
Support from others*	3.2	0.6	0.388	**<0.001**

*others than family/friends

### Associated factors of resilience—selection of the variables

**Fig 1:**
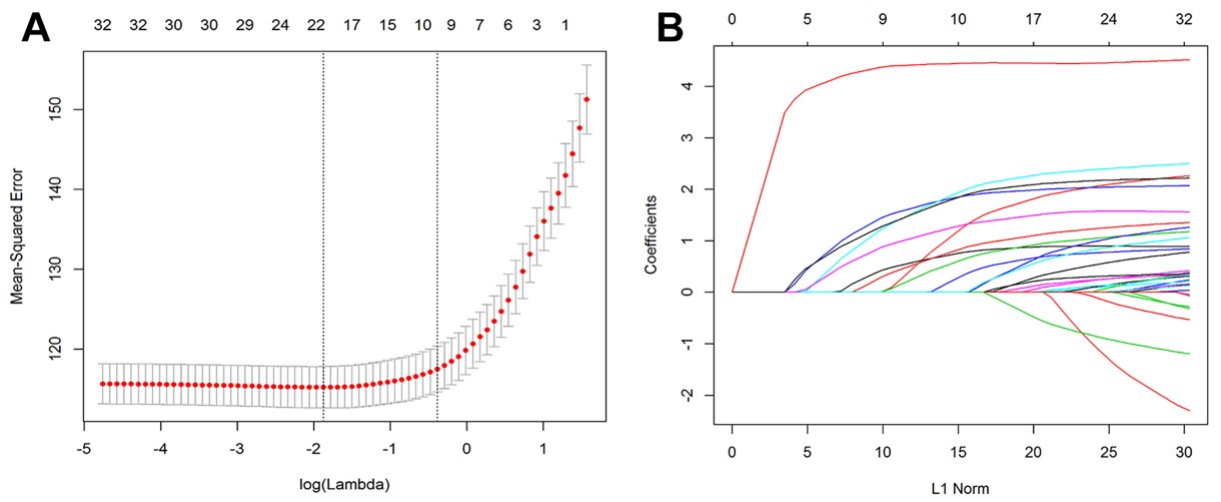
Features selection using the LASSO linear regression model. (A) Optimal parameter (lambda) selection in the LASSO model using tenfold cross-validation via minimum criteria. The partial likelihood deviance (binomial deviance) curve was plotted versus log(lambda). The dotted vertical lines were drawn at the optimal values by using the minimum criteria and the 1 standard error (SE) of the minimum criteria (the 1-SE criteria). (B) LASSO coefficient profiles of the 33 features. A coefficient profile plot was produced against the log(lambda) sequence. Vertical line was drawn at the value selected using tenfold cross-validation, where optimal lambda resulted in 21 features with nonzero coefficients.

Of the participant characteristics, earthquake traumatic experience, and social support, 33 features were reduced to 21 potential factors on the basis of the training set (Fig 1) with non-zero coefficients in the LASSO regression model. These features included sex, father’s education level, relationship with family members, activeness of participation in school activities, academic performance, relationship with classmates, relationship with teachers, health over the last year, undertaking regular physical exercise, receiving psychological counseling, got injured/disabled in the earthquake, witnessed someone else being buried or injury, severe property loss, extreme worry about their own lives, extreme worry about the health of family and friends, feeling guilty about someone else being killed or injured, and support from family/friends/others than family/friends.

### Development and validation of the regression model

**Fig 2:**
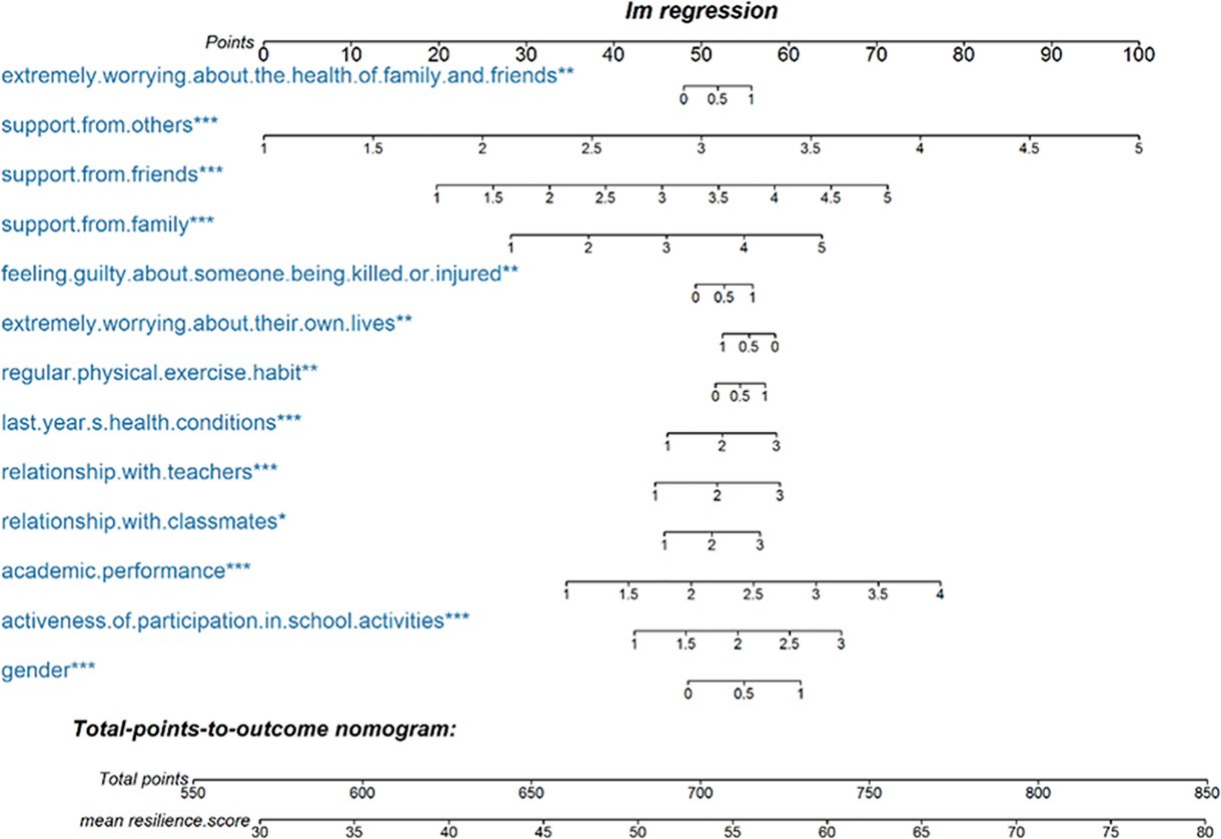
Resilience nomogram. The resilience nomogram was developed using extreme worrying about the health of family and friends, support from others than family/friends, support from friends, support from family, feeling guilty about someone else being killed or injured, extreme worrying about their own lives, regular physical exercise habit, last year health conditions, relationship with teachers, relationship with classmates, academic performance, activeness of participation in school activities, and sex.

Table 4 presents the results of the multiple stepwise linear regression among support from others than family/friends, academic performance, activeness of participation in school activities, support from friends, sex, support from family, relationship with teachers, health over the last year, extreme worry about their own lives, extreme worry about the health of family and friends, relationship with classmates, feeling guilty about someone else being killed or injured, and undertaking regular physical exercise. Among them, support from others than family/friends was the strongest associated factor, while being female and extreme worry about their own lives were negative associated factors. Overall, the model explained 24.61% of the total variance in the participants’ resilience and is presented as a nomogram (Fig 2). Finally, the above linear regression model was applied to the validation set, and the adjusted R^2^ was 0.2731 (p<0.001).

**Table 4:** Multiple stepwise linear regression analysis predicting resilience.

	Partial regression coefficient	Standard error	t	p	R^2^	R^2^adj
Intercept	3.7579	1.6696	2.251	**0.025**	0.2491	0.2461
Support from others than family/friends (original value)	4.4571	0.3700	12.046	**<0.001**		
Academic performance (poor (1), medium (2), good (3), excellent (4))	2.5137	0.2796	8.989	**<0.001**		
Participation in school activities (seldom (1), moderate (2),active (3))	2.0817	0.2729	7.628	**<0.001**		
Support from friends (original value)	2.2659	0.3451	6.566	**<0.001**		
Sex (female (0), male (1))	2.2833	0.3574	6.388	**<0.001**		
Relationship with teachers (poor (1), normal (2), harmonious (3))	1.3079	0.3470	3.770	**<0.001**		
Support from family (original value)	1.6611	0.3381	4.913	**<0.001**		
Health over the last year (poor (1), moderate (2), well (3))	1.1664	0.3081	3.786	**<0.001**		
Extreme worry about the health of family and friends (no (0), yes (1))	1.33763	0.5162	2.591	**0.010**		
Extreme worry about their own lives (no (0), yes (1))	-1.1928	0.3839	-3.107	**0.002**		
Feeling guilty about someone else being killed or injured (no (0), yes (1))	1.1092	0.4486	2.472	**0.013**		
Undertaking regular physical exercise (no (0), yes (1))	0.9585	0.3757	2.681	**0.007**		
Relationship with classmates (poor (1), normal (2), harmonious (3))	0.9656	0.3904	2.473	**0.013**		

## Discussion

Resilience contributes to the recovery of disaster victims. The resilience of Tibetan adolescents after the Yushu earthquake of 2010 has not been properly studied. This cross-sectional survey aimed to examine the current resilience and associated factors in Tibetan adolescent survivors in the hardest-hit area 5 years after the Yushu earthquake. The results suggest that among Tibetan adolescent survivors to the Yushu earthquake of 2010, support from others than family/friends was the strongest positive factor associated with resilience while being female and extreme worry about their own lives were negative factors. These results expand our knowledge regarding resilience in Tibetan adolescent survivors.

Tibetans enjoy good economic policies, so many adults work in their hometowns, and there are few left-behind children. Tibetans also did not implement the one-child policy, so there are fewer only children. Because the Yushu Tibetan area is located in a remote mountainous area, the education received by the older generations was mainly family education and temple education. In this paper, many of the respondents are the first generation of students in their families. Therefore, most of their parents' education background is primary school or below. In the univariable analysis, the scores of mental resilience of parents with college or above degrees were higher than those of parents with other educational levels, but the educational levels of parents did not enter the final regression equation.

In the present study, the CD-RISC score (55.0) of Tibetan adolescent survivors was lower than that (69.6) found in a study by Yu et al. [43], who used the CD-RISC to assess 2914 Chinese community adolescents (mean age of 14.4 years) in Chengdu who had not seriously been affected by the earthquake. On the other hand, the CD-RISC score in the present study was similar with that (51.9) found in a study by Fu et al. [42], who used the CD-RISC to assess 2132 adolescents (mean age of 11.7 years) in earthquake-stricken areas one year after the Wenchuan earthquake. Those differences might be associatedwith differences in the severity of the natural disaster exposure. The Garmezy’s challenge model [47] suggests that too much exposure may make an individual feel miserable and hopeless, and thus unable to cope effectively. Kapoor and Tomar [48] also found that the resilience of junior middle school students in hard-hit areas was lower than students in the more lightly affected areas. Kukihara et al. [49] used the CD-RISC to assess survivors of the 2011 Hiroko earthquake/tsunami/nuclear disaster in Japan nine months after the disaster and found the mean score of CD-RISC was 50.8±19.6. The mean CD-RISC score of the general American population and PTSD patients are 80.4±12.8 and 47.8±19.5, respectively [41].

Being female was found to be a negative factor for resilience in the present study, as supported by Yu et al. [43] and Stratta et al. [50]. Studies have shown that women are more sensitive to stress responses, which in turn can lead to impaired resilience [51]. On the other hand, Ziaian et al. [18] indicated no such negative association of female sex with resilience. This inconsistency may be due to differences in the sources of stress and cultural factors. In Tibetan culture, women play an important role in the family and are often described as “hard workers, obedient servants, and selfless devotees”, but their social participation is minimal [52]. Under the influence of a long-term closed geographical environment and religious culture, Tibetan women have gradually formed a passive psychology of inferiority, obedience, and tolerance, and this has been consolidated and continued in family education, still influencing generation after generation [53]. This may be one reason for the lower resilience score of Tibetan female adolescents in this study. Nevertheless, beyond the cultural differences and the roles of patriarchy and political and socioeconomic factors, it is possible that the small difference between boys and girls might mean nothing on the clinical point of view. This will have to be examined further.

Good health and performing regular physical exercise were found to be positive factors associated with resilience in our study, as supported by previous studies [29,54]. Regular and appropriate physical exercise can maintain and promote physical health, which is a path to resilience because it is related to many characteristics and attributes required for resilience, such as toughness, self-esteem, and self-efficacy [55]. Physical health can also buffer stress-related diseases by optimizing the hormonal stress response system, which helps reduce emotional, physiological, and metabolic responses, and increases positive emotions and happiness, thereby enhances resilience [56]. The present study suggests that physical exercise programs could be designed to help cultivate the resilience of Tibetan adolescents.

The positive relationship between academic performance and resilience found in this study is consistent with findings from de la Fuente et al. [57]. To a certain extent, higher academic performance can represent higher emotional intelligence [58] and the use of direct coping strategies [59]. Other possible reasons are that students in China with good grades are more likely to receive attention from teachers, receive appreciation from their parents and relatives, and have successful experiences, all of which could contribute to the improvement of resilience.

The item ‘activeness of participation in school activities’ was also an important factor for resilience. Of course, social life and school systems between China and the Western countries are very different. The main social place of Chinese middle school students is school, second only to home, their main social role is to study, and they have little contact with society beyond their fellow students, the teachers, and the school staff. Therefore, it might be considered that Chinese adolescents participating in school activities is like engaging in normal activities in a Western context. Returning to normal activities is seen as a way of being resilient [60]. In addition, previous Western studies demonstrated that maintaining a high level of connectivity with school has a protective effect on students who experience a variety of life stressors [61]. Participating actively in various activities could improve their abilities, expand their social networks, increase their sense of belonging as school members, and thus enhance their resilience [48]. Brooks [30] also argues that schools should, as far as possible, provide diverse, meaningful opportunities to cultivate students’ resilience. Accordingly, we also found that having harmonious relationships with classmates and teachers was a positive factor, as supported by Graber et al. [62]. Students having a harmonious relationship with classmates and teachers can feel respect, love, and appreciation from others [63], and are more inclined to have a high level of satisfaction with school [64]. Finally, the quality of the teacher-student relationships affects students’ performance [65], and teachers’ cognition of academic competence is a powerful predictor of students’ efforts and persistence in school [66].

In the present study, all the measured dimensions of social support were positively associated with resilience, consistent with previous studies [67,68]. If properly used, social support is an important resource for individual coping and health [69]. Surprisingly, support from others than family/friends seems to play a more prominent role for Tibetan adolescent survivors, compared to support from friends and family. Since the earthquake, our participants have also experienced the development process from being children to becoming teenagers. During this time, they have received support from family members, caregivers, relatives, friends, teachers, and others to help them cope with the trauma and resulting difficulties.

We found that different earthquake traumatic experiences had different effects on resilience. Notably, ‘feeling guilty about someone else being killed or injured’ and ‘extreme worry about the health of family and friends’ were both positive factors of resilience for Tibetan adolescent survivors, while ‘extreme worry about their own lives’ was a negative factor. The three items were subjective responses to the earthquake and provided a reminder that the subjective responses of individuals to disasters are closely related to mental health, as supported by previous studies [70,71]. It is suggested that the subjective components of earthquake trauma experience should be taken into account during the screening for negative psychological problems and the recruitment of populations for psychological interventions.

This study has several limitations. First, the target population of this study included all the Tibetan secondary school students in Gyegu town who experienced the 2010 Yushu earthquake. Unfortunately, about 1200 potential participants were not included in this study because of the refusal of the principal of one junior high school. Although the general information of students in this school is not obviously different from that of other schools, according to the educational administration department, it might still lead to a bias. Second, the limitation of the cross-sectional study design makes it impossible to determine the causal relationship between variables, as well as the evolution of resilience in time. In addition, no data from before the earthquake are available. Third, the nature of quantitative research makes it difficult to understand how those factors influence the development of Tibetan adolescent survivors’ resilience. Further qualitative research is necessary to explore this. Fourth, due to the specific characteristics of the Chinese culture, social system, and schools, the international generalizability of the conclusions is currently limited. Similar studies should be carried out in various countries. This would provide interesting results about the similarities and differences among different countries/cultures. Fifth, because of the logistics, legal, and ethical complexity of studies dealing with the psychological/psychiatric health of children, the present study did not examine the effect of counseling or psychiatric assessment after the trauma. This is planned for another study. Finally, the Tibetan Buddhist religious was not included in the study because religion is not talked about in school, but the questionnaire was complete and the spiritual item was included. Despite these limitations, this study revealed the associated independent factors of Tibetan adolescent survivors’ resilience. As support from others than family/friends was found to be the most significant associated factor of the participants, and school-level items (e.g., relationship with classmates/teachers, activeness of participation in school activities) were also found to be the associated independent factors, school-based, teacher-mediated intervention programs might be suitable and feasible to improve adolescents' resilience after a natural disaster. The results provide an empirical basis for the selection of the target population for intervention. Future studies should attempt to design, implement, and evaluate training programs for people with relatively low resilience. This study also showed that different disaster experiences had different effects on resilience, and subjective responses of individuals to disasters were independent influencing factors, especially "extreme worry about their own lives". In the process of post-disaster psychological aid, both the screening of negative psychological problems and the selection of psychological intervention populations should consider the subjective components of the earthquake trauma experience, especially those with extreme worry about their own lives should be paid more attention.

Some of the included students were about 7 years of age when the earthquake struck, and they might have a different perception than those who were already adolescents at that time. During the study, we could observe that after the early fear and sadness, these children showed more gratitude, treasure in life, and hope for the future. They are generally more grateful for others' help, and they also want to help others in the future. They cherish their homes and families now. Although the earthquake brought great trauma to Yushu, it also brought in a new life. The construction assistance from all over the country not only made the whole city look brand-new but also increased communication with the outside world. Now Yushu is no longer a remote and backward town in western China, but a famous tourist city in China. The improvement of living conditions and economic conditions increased the hope and expectation for the future in those children.

In conclusion, the findings provide insight into the context-specific factors associated with the levels of resilience among Tibetan adolescent survivors. The results suggest that their resilience needs to be improved despite the fact that many years have passed since the earthquake. This empirical knowledge base can be used to design post-disaster interventions and deploy much-needed resources and services to boost Tibetan adolescent survivors’ resilience. More support should be directed particularly toward females and individuals who experienced the trauma of the earthquake and reported ‘being extremely worried about their own lives’.

## Supporting information

S1 Checklist *PLOS ONE* clinical studies checklist. (DOCX)

S2 Checklist STROBE statement—checklist of items that should be included in reports of observational studies. (DOCX)
